# Tumor-associated macrophages: role in tumorigenesis and immunotherapy implications

**DOI:** 10.7150/jca.49692

**Published:** 2021-01-01

**Authors:** Shunyao Zhu, Ziyi Luo, Xixi Li, Xi Han, Senlin Shi, Ting Zhang

**Affiliations:** 1 College of Pharmaceutical Science, Zhejiang Chinese Medical University, Hangzhou 310053, China.; 2 Xiaoshan Hosptital of Traditional Chinese Medicine, Hangzhou 311201, China.

**Keywords:** tumor-associated macrophages (TAMs), tumor microenvironment, tumor immunotherapy

## Abstract

Tumor-associated macrophages (TAMs) occupy an important position in the tumor microenvironment (TME), they are a highly plastic heterogeneous population with complex effects on tumorigenesis and development. TAMs secrete a variety of cytokines, chemokines, and proteases, which promote the remodeling of extracellular matrix, tumor cell growth and metastasis, tumor vessel and lymphangiogenesis, and immunosuppression. TAMs with different phenotypes have different effects on tumor proliferation and metastasis. TAMs act a pivotal part in occurrence and development of tumors, and are very attractive target to inhibit tumor growth and metastasis in tumor immunotherapy. This article reviews the interrelationship between TAMs and tumor microenvironment and its related applications in tumor therapy.

## Introduction

While containing a large number of malignant epithelial cells, the tumor is also surrounded by surrounding immune cells, neovascularization and its endothelial cells, cancer associated fibroblasts (CAFs), and TAMs surrounded by the extracellular matrix, it forms a unique microenvironment 1.Now, the molecular and biological abnormalities of tumor cells alone cannot fully explain the complex changes in tumorigenesis and development 2,3. Therefore, the effect of tumor microenvironment on tumor progression has become the focus of more and more research 4, 5.

Macrophages are the most inflammatory cells infiltrated into tumors, also known as tumor-associated macrophages. Their content can reach more than 50% of the mass of solid tumors, and they major involved in the inflammatory response of tumors. Studies have shown that treatment methods that target tumor cells alone are not sufficient to treat tumors. Tumor therapy should target tumor cells and their microenvironment as a common target 6. TAMs are significant part of the tumor microenvironment and a very attractive targets for tumor immunotherapy 7-9.

This paper reviews the recent involvement of TAMs in tumorigenesis and its related applications in tumor therapy, in order to find new strategies for treating tumors.

## Origins and characteristics of TAMs

Solid tumors are composed of malignant cells and some non-malignant hematopoietic and mesenchymal cells 10, 11. Macrophages, which are non-malignant cells, are one of the major lymphocytes infiltrating solid tumors. Sometimes the ratio is greater than 50% in tumor tissues. They have the function of phagocytosis and digestion of exogenous armamentarium, including cell fragments and tumor cells to remove detrimental substances 12, 13. Mature macrophages transform into TAMs under appropriate conditions after entering the tumor microenvironment.

The exact origin of TAMs has been controversial. Current research indicates that in mouse models, TAMs are mainly derived from bone marrow monocytes^14^, which are absorbed by inflammatory signals released by cancer cells in primary and metastatic tumors, where they differentiate into TAMs and promote tumor progression^15^, ^16^. However, in tumors such as gliomas and pancreatic cancer, TAMs may also be derived from embryonic macrophages, especially from macrophages deposited in the yolk sac 17. TAM only from embryonic macrophages promotes the growth of formed tumors 18. In both cases, TAMs differentiate into different tumor-related phenotypes in different tumor microenvironments.

**Figure 1:**
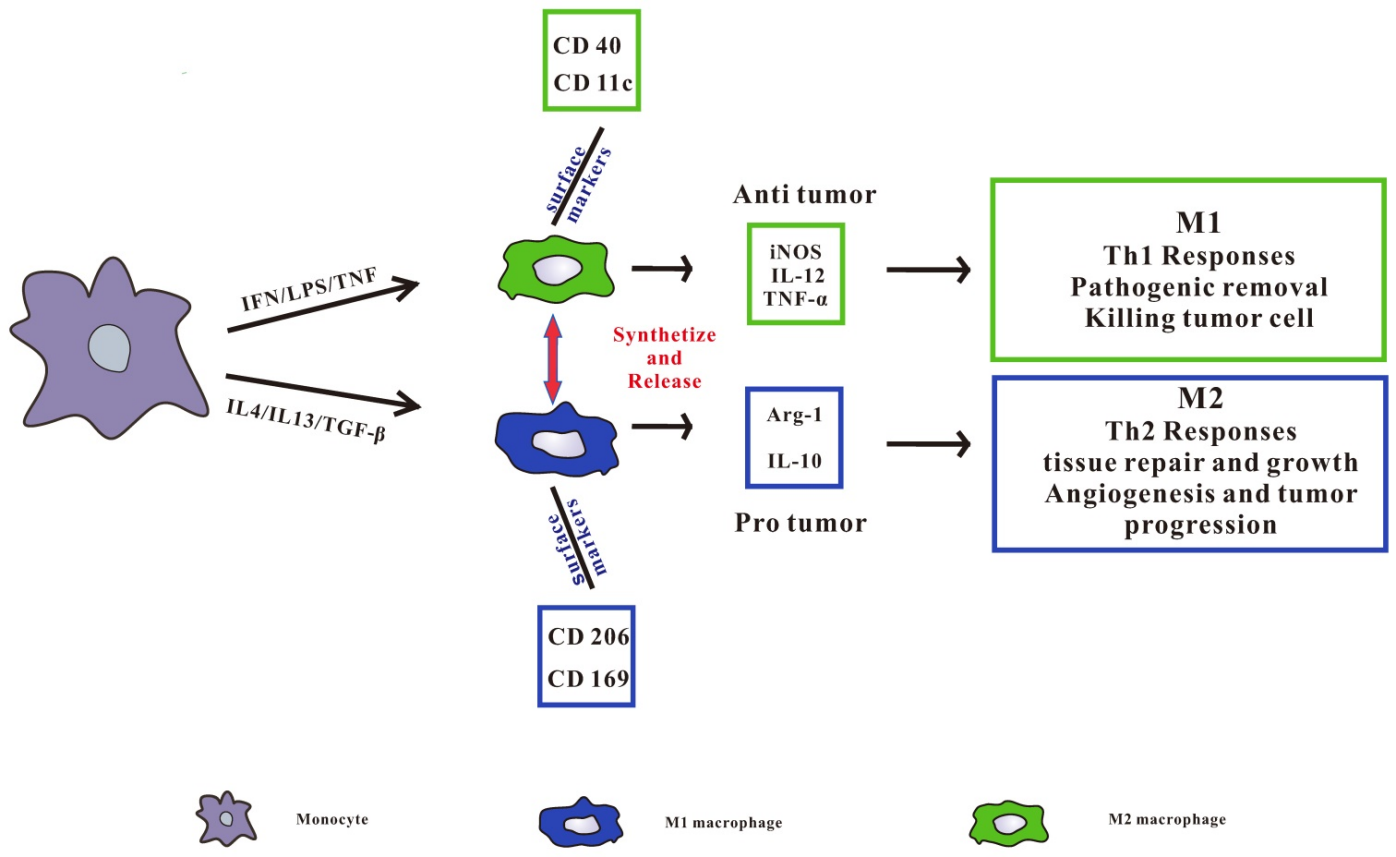
pro-inflammatory M1 type and anti-inflammatory M2 type macrophages. Pro-inflammatory M1 type macrophages are activated by LPS, IFN-γ, or TNF-α. Anti-inflammatory M2 type macrophages are activated by IL4, IL13, or TGF-β. All of the phenotypes synthetize and release a series of different cytokines, chemokines, and receptors, which play different role in tumorigenesis. M1 type macrophages secrete IL-12, iNOS, and TNF-α induce Th1 type immune response, and CD40 and CD11c receptors are highly expressed on the surface. M2 type macrophages secrete Arg-1 and IL-10 to induce Th2 type immune responses and highly express CD206 and CD169 receptors on the surface.

TAMs can be divided into two categories, namely pro-inflammatory M1 type and anti-inflammatory M2 type (Figure 1), in which M2 type macrophages can be further subdivided into M2a, M2b, M2c and M2d subgroups 17, 19. M1 macrophages are also known as classically activated macrophages, which are induced by Interferon-gamma (IFN-γ) 20, other pro-inflammatory cytokines and immune stimulating cytokines such as IL-12 and IL-23^21^. At the same time, it can induce Th1 type immune response with the ability to promote inflammation and anti-tumor immune activity 22. It also removes pathogens, kill tumor cells and play an anti-tumor effect. M2 macrophages are called surrogate activated macrophage. They are induced by IL-4 or IL-4 and IL-13, secrete IL-10, IL-1 receptor antagonists (IL-1RA) and a variety of chemokines, and highly express arginase, mannose receptor, scavenger receptor, etc. M2 macrophages display a low ability to present antigens, mainly induce Th2 type immune responses, and are mainly involved in cell growth, angiogenesis, immunosuppression, tissue repair, and interstitial formation 23, 24, thereby promoting tumor growth.

TAMs are predominantly M2 in most tumors and can be described as M2d subtypes 25. In the course of tumor development, M1 polarized macrophages infiltrated by tumor usually exhibit an IL-12 high IL-10 low phenotype, which promote the immune reaction and cause tumor cell division. During the development of advanced tumors, TAMs usually transform to the M2 phenotype, promote tumor infiltration and metastasis, and create an advantageous microenvironment to promote tumor survival, tumor growth and angiogenesis 26, 27.

## The role and mechanism of TAMs in tumorigenesis and development

### TAMs and the immunosuppressive tumor microenvironment

TAMs are one of the important components of tumor microenvironment 28. Because TME can inhibit immune function, TAMs are generally polarized into M2 macrophage 19. M2 type TAMs are abundant in the tumor stroma, can produce a large number of immunosuppressive chemokines and factors. It can suppress tumor immunity by reducing antigen presentation and blocking T cell function 29. TAMs can restrain the normal course of antigen presentation, such as by secreting cytokines and inflammatory mediators like IL-10, transforming growth factor beta (TGF-β), prostaglandin E_2_ (PGE_2_) and matrix metalloproteinase 7 (MMP-7), thereby making T cells lose the ability to distinguish or even kill tumor cells, which create an immunosuppressive microenvironment 9. Among them, TGF-β and IL-10 are important factors that form the microenvironment of immunosuppressive tumors 30.

TGF-β is a kind of cytokine with immunosuppressive function, and it can inhibit the activity of immune cells such as natural killer (NK) cells, dendritic cells (DCs), and T cells 31. TGF-β can inhibit the NK cell membrane-mediated cytotoxicity-promoting receptors NKp30 and NKG2D, thereby weakening the immune killing of NK cells to tumors 32. The antitumor effect of CD8^+^ T cells is also inhibited by TGF-β. The mechanism is to inhibit the expression of some cell lysing genes, such as granzyme A, granzyme B, IFN-γ and FAS ligand. Further promotes the expansion of the Tregs cell population 33. Moreover, TGF-β reduces DCs transfer and enhance apoptosis, thereby reducing antigen presentation and down-regulating adaptive immune responses, and promoting the differentiation of CD4^+^ T cells to Th2 type 34. TGF-β can also induce tumor cells to overexpress IL-10 to activate Th2 while inhibiting Th1 immune activity, thereby breaking the balance of Th1 / Th2, and ultimately suppressing immune killing of tumor cells 35. Studies show that blocking TGF-β-mediated signaling pathways in the TME could strengthen the killing effect of the immune system on tumors 36, 37.

IL-10 is a versatile cytokine that allows malignant cells to evade immune regulation and promote tumor growth 38. The ability of TAMs to secrete IL-10 is related to another tumor-derived molecule PGE_2_, which regulates TAMs polarization through the EP_2_ and EP_4_ receptors 39. IL-10 is able to restrain the production of pro-inflammatory cytokines by inhibiting the activity of NF-κB, including TNF-α, IL-6 and IL-12^40^. IL-10 could also suppress IFN-γ, which is the principal consideration that stimulates T-cell differentiation and accelerate immune escape. Recent studies have shown that IL-10 has immunostimulatory activity that enhances antitumor immunity 41. When melanoma cells are treated with IL-10 for 48-72 hours can completely inhibit homologous CTL-mediated HLA-A2.1 limited tumor cell up to 100% specific lysis 42. Furthermore, researchers also found that serum IL-10 levels were positively correlated with tumor progression, suggesting that IL-10 is inseparable from the development of tumors 43.

### TAMs and pro-angiogenic effects

TAMs are strictly related to angiogenesis, which is rigid in tumorigenesis and development. As we all know, angiogenesis is essential for the growth and diffusion of malignant tumors. Tumor angiogenesis is the course of generating neovascularization from now available vascular system. The appearance of neovascularization not only provides oxygen and nutrients for tumor growth, but also provides a convenient condition for tumor metastasis 44. There is growing evidence that TAMs make a big difference to adjust angiogenesis. Throughout the process, vascular epithelial growth factor (VEGF), fibroblast growth factor (FGF1), platelet-derived growth factor (PDGF), hepatocyte growth factor (HGF), and placental growth factor (TGF) expressed by TAMs and tumor cells PIGF), matrix metalloproteinases (MMP-9, MMP-2), IL-8, IL-1 and other cytokines play an important synergistic role, of which VEGF is the most important^45^. Studies have shown that triggering M2 polarization of macrophages in lung cancer can enhance the expression of VEGF and thereby advance tumor angiogenesis 46.

Furthermore, TAMs assemble in tumor hypoxic zone be marked by hypoxia tension. TAMs can convey more angiogenic genes based on its adaptation to the hypoxic environment. Hypoxia-inducible factor (HIF)-1 and-2 act a pivotal part in regulating angiogenesis 47. In the hypoxic tumor microenvironment, TAMs enhance the expression of HIF-1and HIF-2^48^, ^49^, and the overexpression of this factor can promote the production of the above cytokines such as VEGF 50, 51.

In a study of PyMT mice, the figure for macrophages in area surrounding tumor was decreased by 43% in PyMT mice treated with doxycycline. Decreasing the number of macrophages through this level can delay tumor progression, reduce tumor production, reduce cancer angiogenesis, and down-regulate the expression of many pro-angiogenic genes 52. Through the study of TAMs in angiogenesis in colon cancer, it was found that TAMs play a vital part in tumors in development of colon cancer in an oxidative stress-dependent manner, thereby enhancing the angiogenic capacity of the tumor microenvironment 53. In the malignant glioblastoma model, M2 type immunosuppressive macrophages promote neovascularization 54. The study found that some TAMs express TIE2 on the surface. These TIE2 ^+^ macrophages usually bind to endothelial cells expressing ANG2 (TIE2 ligand, an endothelial cell-specific angiogenic factor), and they are associated with tumor angiogenesis and tumor ischemia. After the recovery is highly relevant 55.

### TAMs and tumor proliferation, invasion and metastasis

Tumor metastasis is one of the important signs to determine the stages of tumor. Ectopic tumor formation is caused by tumor cell metastasis through blood vessels and lymphatic vessels 56. These all pose huge challenges for tumor treatment, making it difficult to cure and easy to relapse. TAMs can promote the proliferation, invasion and metastasis of tumors 57. It can activate NF-κB and activator of transcription activator STAT3 by expressing inflammatory factors such as TNF-α, IL-6, and IL-11 to enhance tumor cell survival and proliferation 58, 59.

Polarization of TAMs can affect the proliferation, migration, invasion and angiogenesis in primary tumors and metastases. In the study of breast cancer, endometrial cancer and renal cell carcinoma, it was found that the infiltration amount of type M2 TAMs in tumor tissue was positively correlated with tumor cell proliferation. M2 tumor related macrophages advance tumor invasion and metastasis by influencing tumor microenvironment. Studies have confirmed that M2 macrophages secreted EGF, which accelerate epithelial ovarian cancer metastasis by activating EGFR-ERK signaling and inhibiting the deliverance of lncRNA LIMT 60. Epidermal Growth Factor (EGF) secreted by TAMs can make tumor cells form elongated protrusions and enhance their invasion ability. At the same time, Clony stimulating factor-1 (CSF-1) produced by tumor cells can promote macrophages to secrete more EGF, which in turn can promote the production of tumor CSF-1. Significantly increase the invasive power of tumor cells, thereby promoting tumor metastasis 61. In non-small cell lung cancer, TAMs secrete more TGF-β than other macrophage phenotypes and increases SOX9 expression, which strengthens tumor EMT, thereby increases tumor proliferation, migration and invasion 62.

TAMs also produce enzymes (such as metalloproteinases and plasmin) to regulate interstitial metabolism. The activity of these enzymes is related to tumor aggressiveness. For example, MMP-2 is connected with lymph node metastasis and tumor staging in some tumors 63. M2 macrophage-induced deliverance of vascular endothelial growth factor promotes metastatic behavior of Lewis lung cancer cells 64.

### TAMs and Lymphangiogenesis

Lymphangiogenesis is the first step in the general transfer of tumor cells, indicating that the tumor has a poor prognosis. Maintaining the body's homeostasis, normal metabolism and immune response are inseparable from the normal function of the lymphatic system. Experimental and clinical studies have shown that TAMs can markedly advance lymphangiogenesis through cellular autonomic models and paracrine. Paracrine effects include activating lots of pre-existing lymphangiogenic factors.

Studies have found that macrophages are involved in the production of lymphatic vessels in an inflammatory environment. In the study of TAMs on the occurrence and development of ovarian cancer, it was found that TAMs have an impact on the proliferation, migration and capillary-like vessel make up of lymphatic endothelial cells (LEC). TAMs can release VEGF-C, which not only helps tumor angiogenesis, but also promotes tumor lymphangiogenesis 65. Compared with normal ovary, invasive TAMs in malignant ovarian tumor can promote lymphangiogenesis by acting on LEC 66. Overexpression of MMP-2 and MMP-9 in breast cancer can promote lymphangiogenesis and is closely related to lymph node metastasis 67. Maruyama et al. Found that CD11b ^+^ macrophages can accumulate in mouse corneal stroma and express VEGF-C to promote mouse corneal lymphangiogenesis 68.

## Application of TAMs in tumor immunotherapy

**Figure 2:**
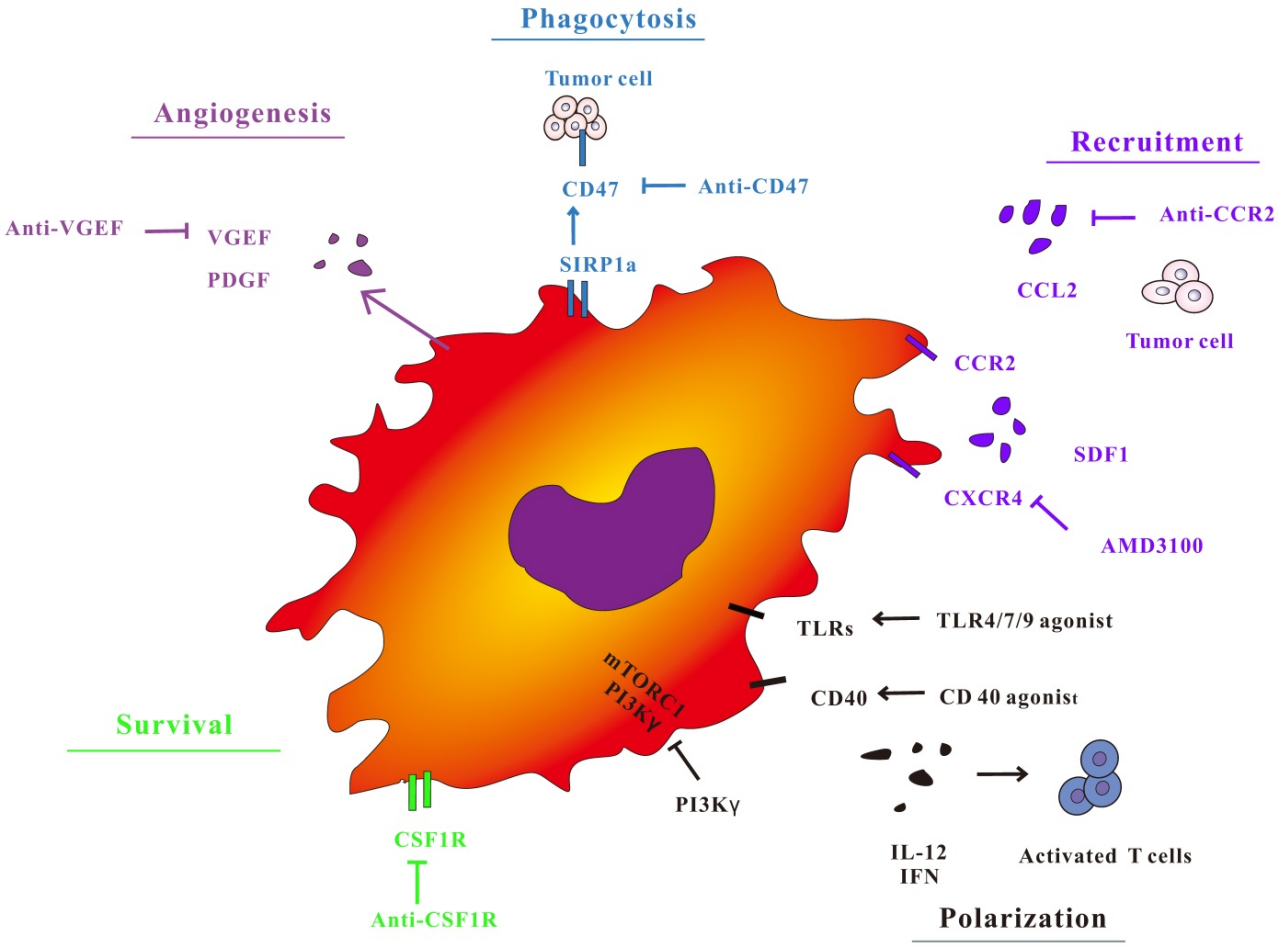
Macrophage targeted cancer treatment strategy. TAM polarization, survival, phagocytosis and angiogenic properties during tumor progression. Targeting key receptors or signaling proteins can inhibit these macrophage properties and inhibit tumor progression.

In view of the importance of TAMs in the regulation of tumor immunity, treatment strategies for macrophages have attracted widespread interest (Figure 2). The treatment of TAMs, including TAMs elimination, changing TAMs phenotype, and improving the antigen presentation function of TAMs not only has a separate anti-tumor effect, but also has a good synergy with immunotherapy methods such as immune checkpoints by animal models and clinical trials.

### Targeting TAMs recruitment depletion and recruitment

One strategy for TAMs consumption is to key off the circulating inflammatory monocyte supply. The circulating monocytes are mobilized from the bone marrow and recruited into the site of inflammation, which highly rely on CCL2-CCR2 signaling. The suppression of CCR2 preserve monocytes in the bone marrow, leading to depletion of circulating cell pools and a reduction in the number of TAMs at the primary and metastatic sites 69-74. The chemokine CCL2 and its receptor CCR2 act a pivotal part in tumor invasion and metastasis by recruiting TAMs. CCL2 infiltrates tumor tissue by recruiting TAMs, secretes VEGF, TGF - β, TNF - α and other cytokines, promotes tumor cell growth and angiogenesis, and also secretes matrix metalloproteinase MMP2, MMP9, participates in the destruction and reconstruction of extracellular matrix, and promotes tumor cell invasion and metastasis. A study of bladder cancer found that lymph node metastasis-related transcripts expressed by tumor cells 1 can recruit heterologous ribonucleoprotein to the CCL2 promoter to activate CCL2 expression. Upregulation of CCL2 recruit macrophages to the tumor and passes VEGF- C promote lymphatic metastasis 75. The increase of CCL2 expression during the carcinogenesis of esophageal squamous cell carcinoma (ESCC) is related to the accumulation of TAMs, both of which indicate a poor prognosis of esophageal cancer. Animal experiments show that CCL2 is blocked-the CCR2 axis is greatly reduced by hindering TAMs recruitment to increase the incidence of tumors, thereby enhancing the anti-tumor efficacy of CD8 (+) T cells in the TME 76. Research progress of adenoid cystic carcinoma of salivary glands (SACC), it was found that the CCL2 derived from SACC cells can activate the expression of its receptor CCR2 in TAMs. The *in vitro* results further indicate that SACC cell-derived CCL2 is involved in TAMs recruitment, M2 polarization and GDNF expression via the CCL2 / CCR2 axis. Treatment of immunodeficient mice with CCR2 antagonists greatly inhibited TAMs infiltration and SACC cell tumorigenicity 77.

CSF-1 is a cytokine that supports the differentiation, proliferation and function of monocytes and macrophages 78. In the preclinical model, a large number of CSF1-CSF1R axes the study 79-81. Inhibiting CSF-1R in a mouse model of glioblastoma can lead to obvious decrease in tumor volume and a significant increase in mouse survival. Although this CSF-1R inhibitory effect does not clear TAMs, it can cause them to be transformed into anti-tumor states regulated by granulocyte-macrophage colony stimulating factor (GM-CSF) 82. At the same time, small molecule inhibitors of CSF1-R have also been shown to consume some TAMs, inhibiting macrophage-mediated immunosuppresssion during tumor recovery, thereby significantly enhancing tumor sensitivity to chemotherapy 83, 84. Inhibition of CSF1R signaling in a mouse model of pancreatic ductal adenocarcinoma can enhance antigen presentation function of macrophages and anti-tumor T cell responses, but these tumor-reactive T cells have PD-1, the expression of PD-1 and other immune checkpoint molecules is increased, which weakens the anti-tumor effect of CSF1R inhibitors. Joint application of immune checkpoint antibodies can enhance its anti-tumor effect. Combination of CSF1R and CXCR2 inhibitor can effectively reduce the number of TAMs and inhibits polymorphonuclear myeloid suppressor cells (PMN-MDSC) in TME, which could delay tumor growth. Nevertheless, the above two drugs used alone in mouse tumor models have no effect on tumor growth 85.

### Remodeling M1 type macrophages to M2 type

Macrophages are functionally plastic, so changing the environmental stimulus under pathological conditions and repolarizing M2 type TAMs into a tumor suppressing phenotype is a potential clinical strategy for cancer treatment. Eliminating or reducing the inducement of M2-type TAMs expression or using certain methods to reverse the phenotype of TAMs have become an important measure to restore the killing ability of macrophages. Reversing the TAMs phenotype can also destroy the tumor immunosuppressive microenvironment, inhibit the formation of tumor blood vessels and lymphatic vessels, and ultimately achieve the purpose of inhibiting tumor proliferation, invasion and metastasis 86. Specifically, in breast tumor models, selective class IIa HDAC inhibitors induce M1 macrophage phenotype, sustain T cell responses and enhance the response to chemotherapy and immune checkpoint blockade 87. Macrophage remodeling may be vital for in the efficacy of tumor cure. It has been shown that activation of PI3Kγ signaling in macrophages can promote immunosuppression of TAMs in lung cancer, pancreatic cancer and melanoma models 88-91. In animal experiments, the pharmacological repression of PI3Kγ leads to macrophage remodeling and enhanced T cell response, either as a unitary drug 92 or in combination with T cell checkpoint suppression 90, 92, 94.

TLR is an important pathogen recognition receptor expressed by TAMs. After TLR3 stimulation, M1 type marker MHCⅡ and costimulatory molecules such as CD86, CD80 and CD40 are up-regulated. In contrast, the expression of the M2-type markers CD206, T-cell immunoglobulin, and mucin domain 3 is reduced. The use of TLR3L in mouse tumors can change M2 type macrophages to M1 type and degenerate tumor growth 95. Ubil^96^ et al. found that Pros1 secreted by tumors inhibited the polarization of M1 type macrophages. Pros1 knockout tumor-bearing mice showed an increase in innate and adaptive immune responses and significantly prolonged survival. The study shows that the Pros1 / TAM interaction may be a new strategy for tumor-mediated immunosuppression. Inhibiting Pros1 and TLR7, 8 is beneficial to promote the polarization of TAM toward M1 and is beneficial to anti-tumor responses.

In a study, it was demonstrated that Lachnum polysaccharide (LEP) can reverse TAM from the tumor-promoting M2 phenotype to the anti-tumor M1 phenotype, thereby enhancing anti-tumor immunity 97. The results showed that glycocalyx simulated nanoparticles could be internalized by TAM through lectin receptor, which resulted in up regulation of immunostimulatory IL-12 and immunosuppressive IL-10. Arginine the down-regulation of enzyme 1 and CCL2 can reverse the function of tumor TAMs to anti-tumor phenotype 98.

When IL-12 was administered to mice with hepatocellular carcinoma, By down regulating STAT3 and its downstream transcription factor c-myc, we can change the functional phenotype of M2 TAMs, so as to reduce the production of tumor cytokines and inhibit the growth of tumors 99. Interleukin (IL) -37 has an anti-tumor effect in hepatocellular carcinoma (HCC) 100. IL-37 promotes the differentiation of TAMs from M2 to M1 subtypes by inhibiting IL-6 / STAT3 signaling. Overexpression of IL-37 in TAMs derived from HCC patients inhibits tumor growth *in vivo*. IL-37 inhibits the M2 polarization of TAMs by regulating the IL-6 / STAT3 pathway, and together inhibits the growth of HCC 101.

### Promote phagocytosis and antigen presentation of TAMs

CD47 / SIRP-α signaling pathway is an important way for tumor immune escape. Almost all human cells express CD47 on the surface, and macrophages can express the CD47 receptor signal-regulating protein α (SIRP-α), and by combining with CD47, they can help macrophages distinguish normal cells from abnormal cells. And tumor cells can express CD47 highly, so as to achieve immune escape 102. The specific antibody of CD47 can prevent the binding of CD47 and SIRP-α, thereby enhancing the immune killing of macrophages against tumors 103. TTI-621 (SIRPαFc) can be combined with CD47 to enhance the phagocytosis of macrophages. In the xenograft model of primary patients with acute myeloid leukemia (AML) the load is significantly reduced 104. Antigen presenting cells, like macrophages and DC express receptors of the TNF receptor superfamily on their surface CD40. Interaction with ligand CD40L (mainly expressed by T cells, basophils and mast cells) can increase the utterance of MHC and the secretion of proinflammatory cytokines, thus encouraging the activation of T cells 105. Glioblastoma multiforme (GBM) is a deadly and highly invasive malignant brain tumor. At present, it has been found that TAMs and microglia are the main cells that promote tumors in the tumor microenvironment. Blocking SIRPα-CD47 signal can induce the phagocytosis of tumor cells by TAMs and microglia, which is effective for various brain tumors including GBM 106.

Leukocyte immunoglobulin-like receptor subfamily B (LILRB), The LILRB family is a class of repressive receptors presented by myeloid cells, and its ligand is MHC class I molecule. MHC class I component β2-microglobulin expressed by tumor cells can straight protect it from being engulfed. This protection is mediated by LILRB1, which is up-regulated on the surface of macrophages, especially TAMs. Blocking MHC class Ⅰ or LILRB1 can enhance the phagocytosis of macrophages after blocking CD47 molecules 107. LILRB2 antagonists inhibit this receptor-mediated activation of tyrosine phosphatase 1/2 and enhance the proinflammatory response. In the company of M-CSF and IL-4, LILRB2 antagonism also inhibits AKT and STAT6 activation. Transcriptome analysis showed that LILRB2 antagonism changed cytoskeletal remodeling, lipid / cholesterol metabolism. Blocking LILRB2 can effectively inhibit the infiltration of myeloid-derived suppressor cells (MDSC) and Treg, and significantly promote the antitumor effect of T cell immune checkpoint inhibitors *in vivo* 108.

Nowadays, anti-PD-1 / PD-L1 therapy has become an important research direction for human cancer immunotherapy. Among them, TAMs can promote the apoptosis of T cells and participate in immunosuppression by expressing PD-L1 on the cell external. Some evidence indicates that TAMs, especially M2-type TAMs, express PD-L1 on their surface 109, promote CD4 + and CD8 + T cell apoptosis through the PD-1 / PD-L1 pathway, and make a big difference in suppressing tumor immune responses 110. PD-L1 is a crucial regulator of the polarization of M2 TAMs. Inhibition of PD-L1 leads to a decrease in M2 markers IL-10 and Arg-1, and an increase in M1 markers IL-12 and TNF-α 111. The study also found that the expression of PD-L1 was increased in circulating monocytes and TAMs induced by tumor cell-derived factors. Blocking PD-1 can reduce the expression of CD47 in TME to restore T cell activity 112, or partially activate DC by TME co-stimulatory molecules to promote an effective anti-tumor T cell response.

### Targeted TAMs therapy

TAMs are an important component of the tumor local immunosuppressive microenvironment. TAMs targeted drug delivery has also attracted widespread attention, which can be roughly divided into three categories: decreasing or depleting TAMs, promoting phagocytosis of TAMs, transforming TAMs into tumor inhibiting macrophages.

Among them, polarization of TAMs is a hot research topic. The targeted nanocarrier that can deliver *in vitro*-transcribed mRNA encoding M1-polarizing transcription factors to reprogram TAMs without causing systemic toxicity. In ovarian cancer, melanoma and glioblastoma models, it has been proved that can reverse the immunosuppressive effect. The tumor support status of TAMs reprograms them into a phenotype that induces anti-tumor immunity and promotes tumor regression 113. Studies have designed, synthesized and evaluated a series of ureido tetrahydrocarbazole derivatives *in vitro* and *in vivo*. Among them, it was found that compound 23a can repolarize TAM from M2 to M1 in a dose-dependent manner both *in vitro* and *in vivo*. More importantly, *in vivo* experiments also show that compound 23a can significantly inhibit tumor growth in LLC mouse models 114. In addition, studies have found that CHA-encapsulated mannosylated liposomes can enhance the immunotherapeutic efficacy of CHA by inducing the transformation from M2 type to M1 type 115.

For reducing or depleting TAMs, the study to achieve the selective elimination of specific M2 TAMs provides the possibility to eliminate TAMs that support cancer while retaining TAMs with anti-tumor potential. EnAd targeting TAMs depletion armed with T cell adaptors provides a powerful treatment that combines direct cancer cell toxicity with the reversal of immunosuppression 116.

It is also very important to promote the phagocytosis of TAMs. Studies have shown that simultaneous inhibition of CSF1-R and SHP2 signaling pathways on the activation and phagocytosis of macrophages may be an effective strategy for macrophage-based immunotherapy. Self-assembled dual inhibitor-loaded nanoparticles (DNTs) target M2 TAMs and simultaneously inhibit the CSF1R and SHP2 pathways. Compared with single drug treatment, it has better phagocytic ability 117.

## Conclusions and perspectives

This review summarizes the research progress of TAMs in cancer treatment related fields in recent years. The source is described, and the role and mechanism in tumorigenesis and development and application in tumor immunotherapy are discussed.

At present, TAM-related tumor immunotherapy has achieved promising results. Research shows that TAMs as the target of anti-tumor treatment strategy mainly focuses on the inhibition of macrophage recruitment and viability, which can significantly improve the efficacy of traditional therapy and immunotherapy. Nevertheless, clinical interest and some implied early test results have not yet brought out the best treatment. Moreover, due to the complex and changeable tumor microenvironment, and the clinical evaluation of immunotherapy mainly focused on single target drugs, its efficacy is limited. Clinical trials have begun to combine the use of different immunotherapy methods, as well as clinical trials that combine immunotherapy with surgery, chemotherapy, radiotherapy, targeted therapy, and photothermal therapy to more accurately treat patients from the perspective of the immune system.

The latest research has shown that macrophages differ markedly from tumor to tumor. Other studies have also analyzed the presence of multiple TAMs subgroups at the molecular level, and the study of macrophages in their primary tumors and their metastasis should be deepened. In recent years, breakthroughs have been made in immunotherapy site therapy. In 2018, it won the Nobel Prize in Physiology or Medicine, and related antibody drugs have been approved for listing. The molecular detection of macrophages has an impact on the progress of tumors, and the research prospects of mechanisms that hinder the response of anti-tumor therapy are also very promising. The reprogramming of TAMs should become the top priority of research. The therapeutic response of M2 type macrophages can be used to direct anti-tumor immune cells to tumors. To attain to this purpose, TAMs can be reshaped into M1 type, which is essential for causing important transduction in TME. The method can also be used in conjunction with other immunotherapy strategies to provide cumulative cancer suppression.

## Abbreviations

TAMs: Tumor-associated macrophages
TME: Tumor microenvironment
CAFs: Cancer associated fibroblasts
IFN-γ: Interferon-gamma
TGF-β: Transforming growth factor beta
PGE_2_: Prostaglandin E_2_
MMP: Matrix metalloproteinase
NK: Natural killer
DCs: Dendritic cells
IL-1RA: IL-1 receptor antagonists
EMT: Epithelial-mesenchymal transition
VEGF: Vascular epithelial growth factor
FGF1: Fibroblast growth factor
PDGF: Platelet-derived growth factor
HGF: Hepatocyte growth factor
HIF: Hypoxia-inducible factor
EGF: Epidermal growth factor
CSF-1: Colony stimulating factor-1
LEC: Lymphatic endothelial cells
CCR2: Chemokine (C-C motif) receptor 2
CCL2: Chemokine (C-C motif) ligand 2
ESCC: Esophageal squamous cell carcinoma
SACC: Salivary adenoid cystic carcinoma
LEP: Lachnum polysaccharide
HCC: Hepatocellular carcinoma
AML: Acute myeloid leukemia
GBM: Glioblastoma multiforme
LILRB: Leukocyte immunoglobulin-like receptor subfamily B
